# Impairment of Nitric Oxide Synthase but Not Heme Oxygenase Accounts for Baroreflex Dysfunction Caused by Chronic Nicotine in Female Rats

**DOI:** 10.1371/journal.pone.0098681

**Published:** 2014-05-28

**Authors:** Mohamed A. Fouda, Hanan M. El-Gowelli, Sahar M. El-Gowilly, Laila Rashed, Mahmoud M. El-Mas

**Affiliations:** Department of Pharmacology and Toxicology, Faculty of Pharmacy, Alexandria University, Alexandria, Egypt; Temple University, United States of America

## Abstract

We recently reported that chronic nicotine impairs reflex chronotropic activity in female rats. Here, we sought evidence to implicate nitric oxide synthase (NOS) and/or heme oxygenase (HO) in the nicotine-baroreflex interaction. Baroreflex curves relating changes in heart rate to increases (phenylephrine) or decreases (sodium nitroprusside) in blood pressure were generated in conscious female rats treated with nicotine or saline in absence and presence of pharmacological modulators of NOS or HO activity. Compared with saline-treated rats, nicotine (2 mg/kg/day i.p., for 14 days) significantly reduced the slopes of baroreflex curves, a measure of baroreflex sensitivity (BRS). Findings that favor the involvement of NOS inhibition in the nicotine effect were (i) NOS inhibition (*N*_ω_-Nitro-L-arginine methyl ester, L-NAME) reduced BRS in control rats but failed to do so in nicotine-treated rats, (ii) L-arginine, NO donor, reversed the BRS inhibitory effect of nicotine. Alternatively, HO inhibition (zinc protoporphyrin IX, ZnPP) had no effect on BRS in nicotine- or control rats and failed to reverse the beneficial effect of L-arginine on nicotine-BRS interaction. Similar to female rats, BRS was reduced by L-NAME, but not ZnPP, in male rats and the L-NAME effect was not accentuated after concomitant administration of nicotine. Baroreflex dysfunction caused by nicotine in female rats was blunted after supplementation with hemin (HO inducer) but not tricarbonyldichlororuthenium(II) dimer (CORM-2), a carbon monoxide (CO) releasing molecule, or bilirubin, the breakdown product of heme catabolism. The facilitatory effect of hemin was abolished upon simultaneous treatment with L-NAME or 1H-[1], [2], [4] oxadiazolo[4,3-a] quinoxalin-1-one (inhibitor of soluble guanylate cyclase, sGC). The activities of HO and NOS in brainstem tissues were also significantly increased by hemin. Thus, the inhibition of NOS, but not HO, accounts for the baroreflex depressant of chronic nicotine. Further, hemin alleviates the nicotine effect through a mechanism that is NOS/sGC but not CO or bilirubin-dependent.

## Introduction

Cigarette smoking is among the top ten contributors of morbidity and mortality in the world [1]. It predisposes to several cardiovascular diseases including hypertension, coronary heart disease and myocardial infarction [2]. Because these cardiovascular events are replicated with nicotine alone, it is concluded that the nicotine content of tobacco is largely responsible for the detrimental cardiovascular actions of cigarette smoking [3]. Arterial baroreceptor dysfunction, among several other factors, contributes to increased vulnerability of smokers to cardiovascular risk [4]. Nicotine diminishes the baroreflex gain through direct interaction with central mechanisms integrating the baroreceptor input into autonomic responses [5] or via reducing arterial compliance and stretch receptor responsiveness [6]. In a recent study, we presented the first experimental evidence that chronic nicotine dose-dependently attenuated reflex heart rate (HR) responses in female rats through a mechanism that involved the inhibition of the estrogen-mediated vagal facilitation [7].

Nitric oxide (NO) variably affects the autonomic regulation of cardiovascular function [8], [9]. The inhibition of NOS activity in central medullary areas attenuates reflex bradycardic responses and this effect is reversed in presence of the nitric oxide synthase (NOS) substrate L-arginine [10]. The gain of the renal sympathetic nerve activity is increased after systemic NOS inhibition in rabbits [11] and rats [12]. Like NOS/NO signaling, the importance of the heme oxygenase (HO)/carbon monoxide (CO) system in the regulation of the baroreflex-mediated HR control has been established. For example, the inhibition of HO activity in the nucleus of the solitary tract (NTS), the brainstem neuronal pool that receives and integrates baroreceptor afferents [13], attenuates the baroreceptor reflex [14] and reduces the depressor and bradycardic responses elicited by glutamate in the NTS [15]. Also, reduced baroreceptor activity along with increased blood pressure, myogenic vasoconstriction, and increased responsiveness to vasopressor agents have been demonstrated after systemic HO inhibition [16].

Growing knowledge has been recognized concerning facilitatory crosstalk between gaseous products of NOS (NO) and HO (CO) pathways and the interplay of their actions in the understanding of vascular control and disease states [17]. The activation of sGC and subsequent accumulation of cGMP is a common signaling pathway that mediate the biological effects of both NO and CO [18]. NO increases the expression of HO-1 in endothelial and vascular smooth muscle cells [19]. Unlike this seemingly synergistic interaction between NO and CO, contrasting biological effects for the two systems have also been described [20]. Further, Achouh et al. [21] demonstrated that the relaxant effect of CO in the human internal thoracic artery is independent of the endothelium and is preserved following NOS inhibition.

Despite the importance of NOS/HO signaling in the control of arterial baroreflexes as detailed earlier, there has been no study to our knowledge that evaluated whether the interruption of these pathways or their mutual interactions contributes to the nicotine-evoked baroreflex dysfunction. Accordingly, in this study we report on the effect of pharmacologic inventions that alter (inhibit or facilitate) NOS or HO activity on the nicotine-baroreflex interaction. The study was then extended to test the hypotheses that (i) NO/CO crosstalk modulates the baroreflex action of nicotine, and (ii) bilirubin, a byproduct of heme catabolism [22], participates in the evoked baroreflex response. Experiments were undertaken in conscious female rats pre-instrumented two days earlier with indwelling femoral catheters for the measurement of blood pressure (BP) and i.v. drug administration. A dose of 2 mg/kg/day of nicotine was given for two weeks, which has been shown in our previous studies to produce plasma cotinine levels [7] similar to those achieved in humans after moderate cigarette smoking [23], [24].

## Materials and Methods

Adult female Wistar rats (200–250 g; Faculty of Pharmacy animal facility, Alexandria University, Alexandria, Egypt) were used in this study. All experiments were approved by the institutional Animal Care and Use Committee (IACUC, Faculty of Pharmacy, Alexandria University, Egypt, permit number 10–45). Surgery was performed under thiopental anesthesia, and all efforts were made to minimize suffering.

### Intravascular Cannulation

The method described in our previous studies [25], [26] was adopted. In brief, rats were anesthetized with thiopental (50 mg/kg i.p.). Catheters (each consisting of 5 cm polyethylene-10 tubing bonded to 15 cm polyethylene-50 tubing) were placed in the abdominal aorta and vena cava via the femoral artery and vein for measurement of BP and i.v. administration of drugs, respectively. The polyethylene-10 portion was used for the intravascular segment of the catheter. The arterial catheter was connected to a BP transducer (model P23XL; Astro-Med, West Warwick, RI) that was attached through MLAC11 Grass adapter cable to a computerized data acquisition system with LabChart-7 pro software (Power Lab 4/35, model ML866/P; AD Instruments Pty Ltd., Castle Hill, Australia). The LabChart-7 Pro software computes the HR by applying the cyclic measurement function, which is a channel calculation that analyzes periodic blood pressure waveforms in realtime. Data of the detected cycles are displayed as a continuous data trace for HR in another channel of the data acquisition system. Finally, catheters were tunneled subcutaneously, exteriorized at the back of the neck between the scapulae, flushed with heparin (0.2 ml; 100 U/ml), and plugged by stainless steel pins. The incisions were closed by surgical clips. Each rat received an i.m. injection of 60,000 U of benzathine penicillin. Experiments started 2 days later in conscious rats. This time period has been shown to be adequate for recovery from surgery and restoration of normal rat activity [25], [27], [28].

### Measurement of brainstem HO activity

The method described in our previous studies was adopted [29]. Briefly, brainstem tissues were homogenized in 10 mM Tris-HCl buffer (pH 7.6) containing 250 mM sucrose and 0.4 mM phenylmethylsulfonyl fluoride. The homogenate was centrifuged at 800 *g* for 10 min and then at 13,500 *g* for 20 min to produce the mitochondrial pellet. The supernatant was incubated at 37°C for 1 hr in 0.5 ml of 0.1 M phosphate buffer saline (pH 7.4) containing heme (50 µM), rat liver cytosol (5 mg/ml), MgCl2 (2 mM), glucose-6- phosphate dehydrogenase (1 U), glucose-6-phosphate (2 mM), and reduced nicotinamide adenine dinucleotide phosphate (0.8 mM). The reaction was stopped by placing the tubes on ice and the bilirubin generated was extracted by chloroform and quantified with a scanning spectrophotometer at 463 and 520 nm.

### Measurement of brainstem NOS activity

Brainstem tissues were homogenized in ice-cold 25 mM Tris buffer (pH 7.6) containing 0.5 mM D,L-dithiothreitol, 10 mg/ml leupeptin, 10 mg/ml pepstatin, 10 mg/ml aprotinin, 1 mM phenylmethylsulphonyl fluoride. The homogenate was centrifuged at 800 *g* for 10 min. NOS activity in the supernatant was determined by the L-[^3^H]-citrulline generation method according the the manufacturer's instructions (Cayman chemical, Michigan, USA) and as described elsewhere [30]. A volume of 10 µl crude homogenate was added to 25 mM Tris containing 1 mM D,L-dithiothreitol, 30 mM 5,6,7,8- tetrahydro-L-biopterin dihydrochloride, 10 mM flavin adenine dinucleotide, 0.5 mM inosine, 0.5 mg/ml BSA, 0.1 mM CaCl_2_, 10 mM L-arginine and 50 nM L-[^3^H]-arginine, pH 7.6. The final incubation volume was 100 µl. NADPH (10 µl) was added to a final concentration of 0.75 mM and maintained at 37°C for 30 min. The reaction was stopped by the addition of 400 µl cold 0.1 mM HEPES, 10 mM EGTA and 0.175 mg/ml L-citruline, pH 5.5. The reaction mixture was decanted into a 2 ml column packed with ion exchange resin (Na^+^ form) and eluted with 1.2 ml water. L-[^3^H]-citruline was quantified by liquid scintillation spectroscopy. The retention of L-[^3^H]-arginine in this process was greater than 98%.

### Protocols and experimental groups

#### Role of NOS/NO signaling in the baroreflex depressant effect of nicotine in female rats

A total of six groups of conscious female rats (n = 6–8 each) were used in this experiment to investigate the effect of NOS inhibition (L-NAME) or induction (L-arginine) on the nicotine-evoked baroreflex dysfunction. Three rat groups received i.p. nicotine at a dose of 2 mg/kg/day for 14 days, while the other three groups received equal volume of saline. Intravascular cannulation was performed, as described earlier, on day 12 and baroreflex responsiveness was measured 2 days later in conscious animals. On the experiment day (day 14), the arterial catheter was connected to the pressure transducer and Power Lab data acquisition system for the measurement of BP and HR, and an approximately 30-min period of hemodynamic stabilization was allowed. Afterward, baroreflex testing was performed in all rat groups (nicotine or saline-treated) 15 min after treatment with i.v. L-NAME (10 mg/kg), L-arginine (100 mg/kg) or saline.

Another group of nicotine-treated female rats (n = 7) was utilized to investigate whether the HO-1 inhibition by ZnPP abrogates baroreflex changes caused by L-arginine. Rats in this group were injected with nicotine (2 mg/kg/day) for 14 consecutive days. Baroreflex curves for phenylephrine (PE) and sodium nitroprusside (SNP) were then established 15 min after i.v. administration of L-arginine (100 mg/kg) plus ZnPP (10 mg/kg).

Baroreflex sensitivity was assessed by a modified Oxford (vasoactive) method [31] where changes in HR derived from BP waveforms were correlated to changes in MAP caused by vasopressor and vasodepressor agents [7], [10]–[12], [25]. This method measures the bradycardic or tachycardic responses to reciprocal peripherally-mediated BP changes evoked by bolus i.v. injections of randomized doses of PE or SNP (1–16 µg/kg, every 5 min). The relationship between the peak changes in mean arterial pressure (MAP) evoked by PE or SNP and associated reciprocal changes in HR was assessed by regression analysis for individual animals. The regression coefficient (slope of the regression line, BRS_PE_ and BRS_SNP_) expressed as beats/min/mmHg was taken as an index of baroreflex responsiveness. Alternatively, BRS was also assessed by correlating peak changes in heart period (R-R intervals, reciprocal of HR in milliseconds) to changes in MAP evoked by the vasoactive agents [14], [32]. The regression coefficients (slopes of the regression lines expressed in ms/mmHg) were taken as an index of BRS.

To eliminate any role for the altered MAP responsiveness to PE or SNP caused by the modulators of NOS activity in the associated BRS changes, HR responses to similar increases (PE, ∼40 mmHg) or decreases (SNP, ∼−20 mmHg) in MAP were computed for individual rats, regardless of the doses of vasoactive agents employed. The ratio ΔHR/ΔMAP was taken as a measure of BRS as described in our previous studies [7]. The data obtained from this experiment are included in file S1 and in figure S1.

To determine whether the interaction of nicotine with reflex tachycardic activity was SNP-specific, two more groups of female rats (n = 6 each) was used to determine the effect of chronic nicotine (2 mg/kg/day for 2 weeks) on reflex tachycardic responses elicited by hydralazine (1 mg/kg i.v.). Peak changes in MAP and HR caused by hydralazine in rats pretreated with saline or nicotine were measured and used for the calculation of BRS_hydralazine_ (ΔHR/ΔMAP). In a third group of nicotine-treated rats (n = 7), BRS_hydralazine_ was evaluated after the i.v. administration of 100 mg/kg L-arginine.

#### Role of HO/CO signaling in the baroreflex depressant effect of nicotine in female rats

The effect of HO-1 inhibition (ZnPP) or induction (hemin) on the baroreflex depressant effect of nicotine was investigated in five groups of nicotine (2 mg/kg/day for 14 days, n = 6–8)-treated female rats. In these rats, baroreflex testing was performed after i.v. treatment with (i) ZnPP (10 mg/kg, 15 min), (ii) hemin (15 mg/kg, 30 min), (iii) hemin plus L-NAME (10 mg/kg), (iv) hemin plus 1H-[1], [2], [4] oxadiazolo[4,3-a] quinoxalin-1-one (ODQ, 2 mg/kg, 30 min), or (v) CORM-2 (10 mg/kg, 3 min). A sixth group of rats (n = 8) was used to test the baroreflex sensitivity (BRS) effect of i.v. ZnPP after 14 days of saline administration. The doses of ZnPP, hemin, or CORM-2 were selected based on reported studies [33], [34]. As controls, two more groups of female rats were employed that received saline for 14 consecutive days. Baroreflexes were measured in these rats after the administration of hemin of CORM-2. For determination of HO or NOS activity, brains were collected from rats treated chronically with nicotine or saline in the absence or presence of hemin. Brainstem tissues were dissected and stored at −80°C till used for the measurement of HO or NOS activity as described earlier.

#### Effect of bilirubin on the nicotine-baroreflex interaction in female rats

Because the preceding experiment demonstrated the ability of hemin to abrogate baroreflex dysfunction caused by nicotine, the possibility was investigated that bilirubin, a byproduct of heme degradation [22], contributed to the favorable BRS action of hemin. Two groups of female rats were employed and treated with i.p. nicotine (2 mg/kg/day, n = 7) or saline (n = 7) for 14 days. Baroreflex curves were generated in these rats before and 15 minutes after i.v. administration of bilirubin (5 mg/kg) [35]. For the measurement of blood bilirubin, two blood samples were collected into heparinized tubes before and 15 min after the administration of bilirubin. Similarly, blood samples were also collected from the hemin group employed in the preceding experiment. The collected blood was centrifuged at 1200×g for 10 minutes. The plasma was aspirated and stored −80°C till analyzed colorimetrically for the total bilirubin [35].

#### Effect of nicotine on baroreflex gain in male rats

This experiment investigated whether the interaction of chronic nicotine with reflex HR control seen in female rats and its modulation by the NOS/HO signaling could be replicated in male rats. The experimental groups employed in this experiment and the data obtained are outlined in the file S1 and figure S2.

Drugs. Bilirubin, CORM-2, hemin, L-arginine, L-NAME, ODQ, nicotine, phenylephrine hydrochloride, sodium nitroprusside, Zinc protoporphorin IX, hydralazine hydrochloride (Sigma, St. Louis, MO, USA), thiopental (Thiopental, Biochemie GmbH, Vienna, Austria), povidone-iodine solution (Betadine; Nile Pharmaceutical Co., Cairo, Egypt), and benzathine penicillin (Penicid; Cid Pharmaceutical Co., Cairo, Egypt) were purchased from commercial vendors. CORM-2 was dissolved in dimethyl sulfoxide (0.1% solution). ZnPP was prepared daily by dissolving 10 mg in 1 ml of 50 mmol Na_2_CO_3_. Hemin (15 mg) or bilirubin (5 mg) was prepared daily by dissolving in 0.1 N NaOH. The solutions were than titrated to pH 7.4 with 0.1 N HCl and the volumes were completed to 2.5 ml with phosphate buffer. Solutions of bilirubin, hemin, and ZnPP were kept in amber glass vials wrapped in aluminum foil to protect them from light [36].

#### Statistical Analysis

Values are expressed as means ± S.E.M. Multiple comparisons were analyzed by one way analysis of variance (ANOVA) followed by the Newman-Keuls post-hoc test. The analysis was performed using GraphPad Prism, software release 3.02. Probability levels less than 0.05 were considered significant.

## Results

Baseline values of MAP and HR in rats treated with i.p. nicotine (2 mg/kg/day, for 14 days) or saline prior to i.v. drug treatments were not statistically different (Table 1).

**Table 1:** Baseline values of mean arterial pressure (MAP, mmHg) and heart rate (HR, beats/min) in different female rat groups.

Group	MAP	HR
Saline/saline	104.92±3.92	376.54±6.94
Saline/L-NAME	103.71±3.61	351.02±17.15
Saline/ZnPP	106.15±5.66	384.5±20.72
Saline/bilirubin	91.95±5.2	388.5±14.23
Nicotine/saline	98.8±4.19	377.78±6.51
Nicotine/L-NAME	105.53±4.78	376.33±15.79
Nicotine/L-arginine	102.94±3.68	366.86±11.66
Nicotine/ZnPP	103.96±2.95	359.67±17.13
Nicotine/hemin	94.76±3.43	337.73±7.36
Nicotine/L-arginine/ZnPP	95.66±1.64	335.44±7.79
Nicotine/hemin/L-NAME	99.1±3.84	362.46±8.42
Nicotine/CORM-2	102.32±3.92	359.95±11.11
Nicotine/bilirubin	101.96±3.51	352.7±21.53

Values are expressed as means ± SEM.

### Role of NOS/NO signaling in the baroreflex depressant effect of nicotine in female rats

**Figure 1:**
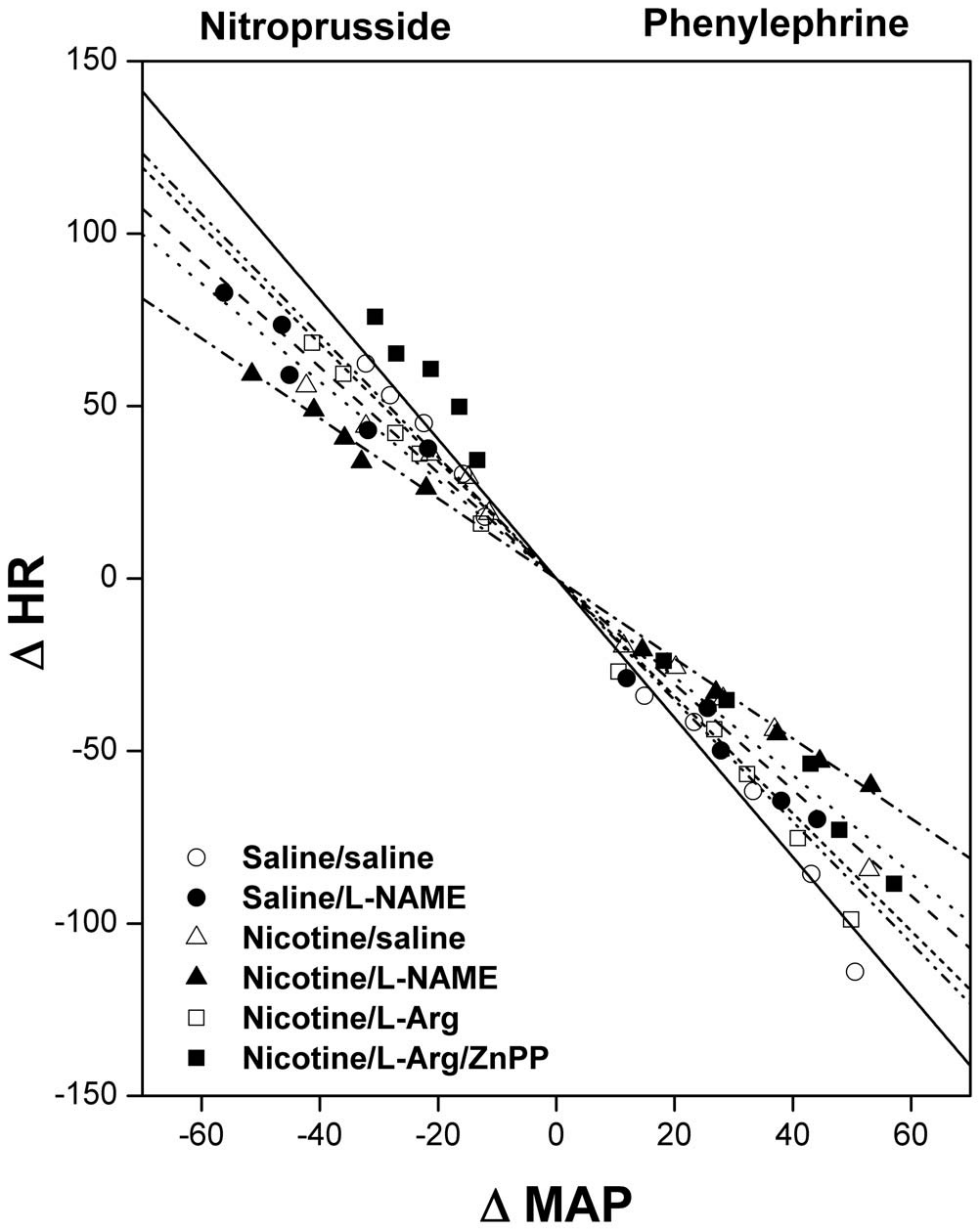
Baroreflex curves relating changes in mean arterial pressure (MAP) elicited by sodium nitroprusside and phenylephrine to reciprocal changes in heart rate (HR) in conscious female rats treated for 14 days with nicotine (2 mg/kg/day, i.p.) or its vehicle (saline) in the absence or presence of L-NAME (NOS inhibitor) or L-arginine (NOS substrate). The effect of HO inhibition by ZnPP on the facilitatory baroreflex action of L-arginine is also shown. Values are means ± SEM of 6–8 observations.

**Figure 2:**
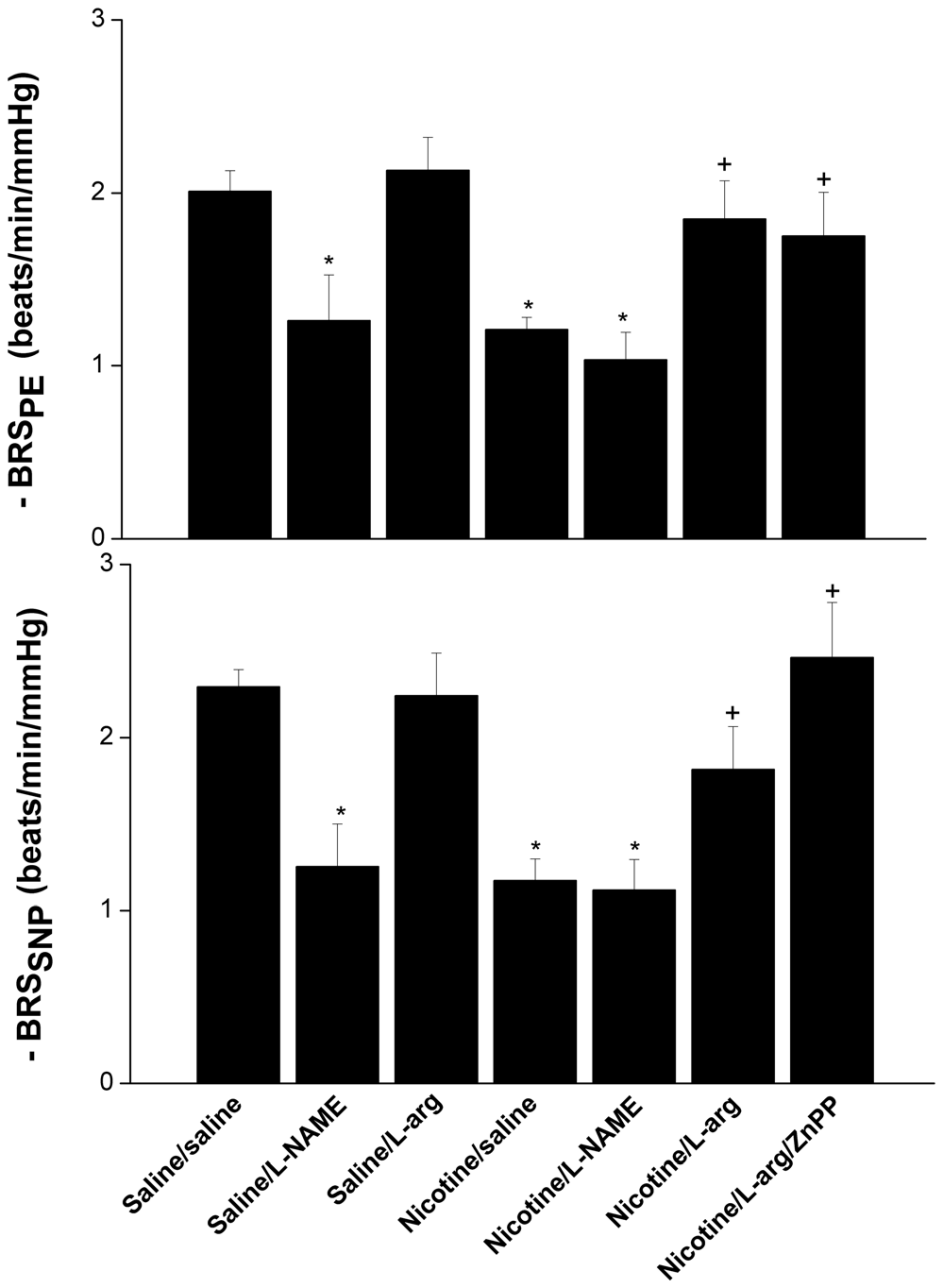
Effect of L-NAME (NOS inhibitor) or L-arginine (NOS substrate) on baroreflex sensitivity (slopes of baroreflex curves) measured by sodium nitroprusside and phenylephrine in conscious female rats treated for 14 days with nicotine (2 mg/kg/day, i.p.) or its vehicle (saline). The effect of HO inhibition by ZnPP on baroreflexes in nicotine/L-arginine-treated rats is also shown. Values are means ± SEM of 6–8 observations. ^*^P<0.05 vs. control, ^#^P<0.05 vs. nicotine.

Figures 1 and 2 depict the effects of NOS inhibition (L-NAME) or induction (L-arginine) on baroreflex curves relating increases (phenylephrine, 1–16 µg/kg) and decreases (SNP, 1–16 µg/kg) in MAP to reciprocal changes in reflex HR responses in female rats treated chronically with nicotine or saline. Nicotine-treated rats exhibited upward and downward shifts in the PE and SNP baroreflex curves, respectively, suggesting attenuated reflex chronotropic responses by nicotine (Fig. 1). The slopes of baroreflex curves (regression coefficients), which represented the BRS_PE_ and BRS_SNP_, were also significantly decreased in nicotine compared with control (saline-treated) rats (Fig. 2). Similar reductions in BRS were caused by nicotine when changes in heart period (R-R intervals) and MAP were correlated. Compared with saline-treated values, nicotine caused significant reductions in BRS_PE_ (2.1±0.2 vs. 1.1±0.2 ms/mmHg) and BRS_SNP_ (0.9±0.1 vs. 0.4±0.1 ms/mmHg). The correlation coefficients of all regression lines relating changes in HR to changes in MAP were highly significant (P<0.005) and ranged from 0.89 to 0.99.

Similar upward and downward shifts in baroreflex curves of PE and SNP, respectively (Fig. 1) and reductions in BRS (Fig. 2) were seen when control (saline-treated, 2 weeks) rats were treated with the NOS inhibitor L-NAME (10 mg/kg, i.v.). The reductions caused by L-NAME in reflex HR responses were similar to those caused by nicotine (Figs. 1 and 2). By contrast, no further decreases in reflex HR responses (Fig. 1) or BRS (Fig. 2) were observed when i.v. L-NAME was administered to nicotine-treated rats. On the other hand, the depressant effect of nicotine on reflex chronotropic responses disappeared when rats were treated intravenously with the NOS substrate L-arginine (100 mg/kg) (Figs. 1 and 2). This beneficial effect of L-arginine was preserved when the nicotine-treated rats were treated concomitantly with ZnPP (HO-1 inhibitor, 15 mg/kg) (Figs. 1 and 2). On the other hand, L-arginine caused no changes in BRS_PE_ or BRS_SNP_ when given to saline-treated rats (Fig. 2). Similar to its effect on BRS_SNP_, nicotine also caused significant reductions in the BRS (ΔHR/ΔMAP) tested by hydralazine (1 mg/kg) from −5.35±0.40 to −3.57±0.28 beats/min/mmHg. The attenuating effect of nicotine on BRS_hydralazine_ disappeared in female rats treated intravenously with 100 mg/kg L-arginine (−5.12±0.50 beats/min/mmHg).

### Role of HO/CO signaling in the baroreflex depressant effect of nicotine in female rats

**Figure 3:**
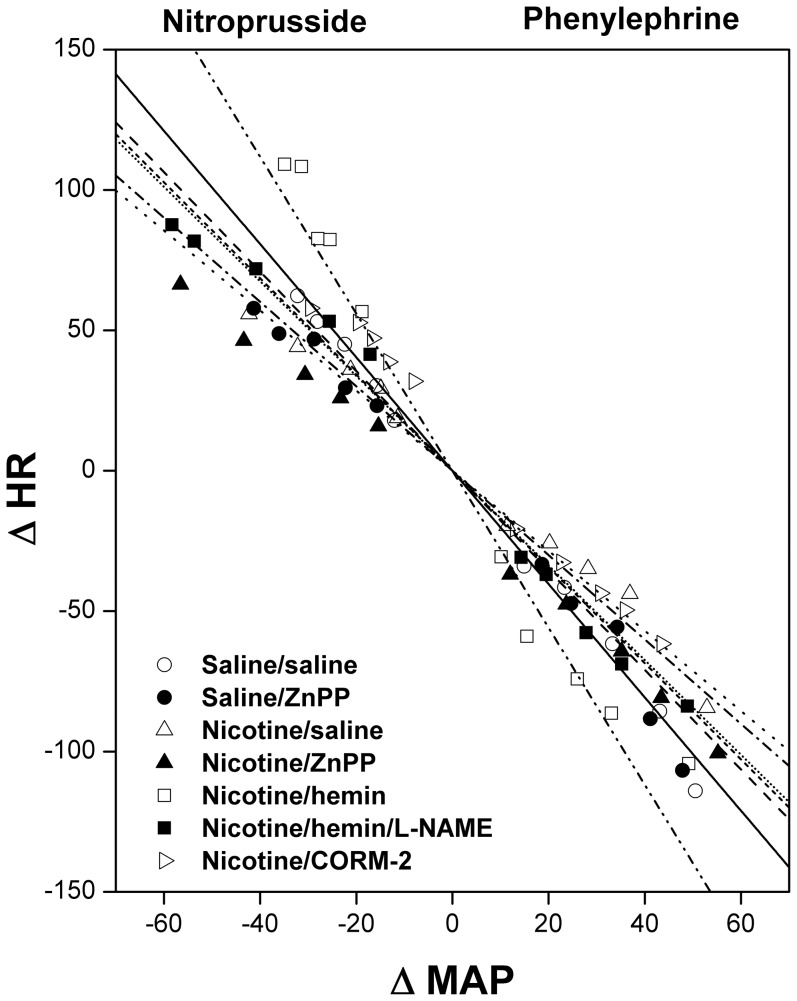
Baroreflex curves relating changes in mean arterial pressure (MAP) elicited by sodium nitroprusside and phenylephrine to reciprocal changes in heart rate (HR) in conscious operated female rats treated for 14 days with nicotine (2 mg/kg/day, i.p.) or its vehicle (saline) in the absence or presence of ZnPP (HO inhibitor), hemin (HO inducer), or CORM-2 (CO releasing molecule). The effect of NOS inhibition by L-NAME on the facilitatory baroreflex action of hemin is also shown. Values are means ± SEM of 6–8 observations.

**Figure 4:**
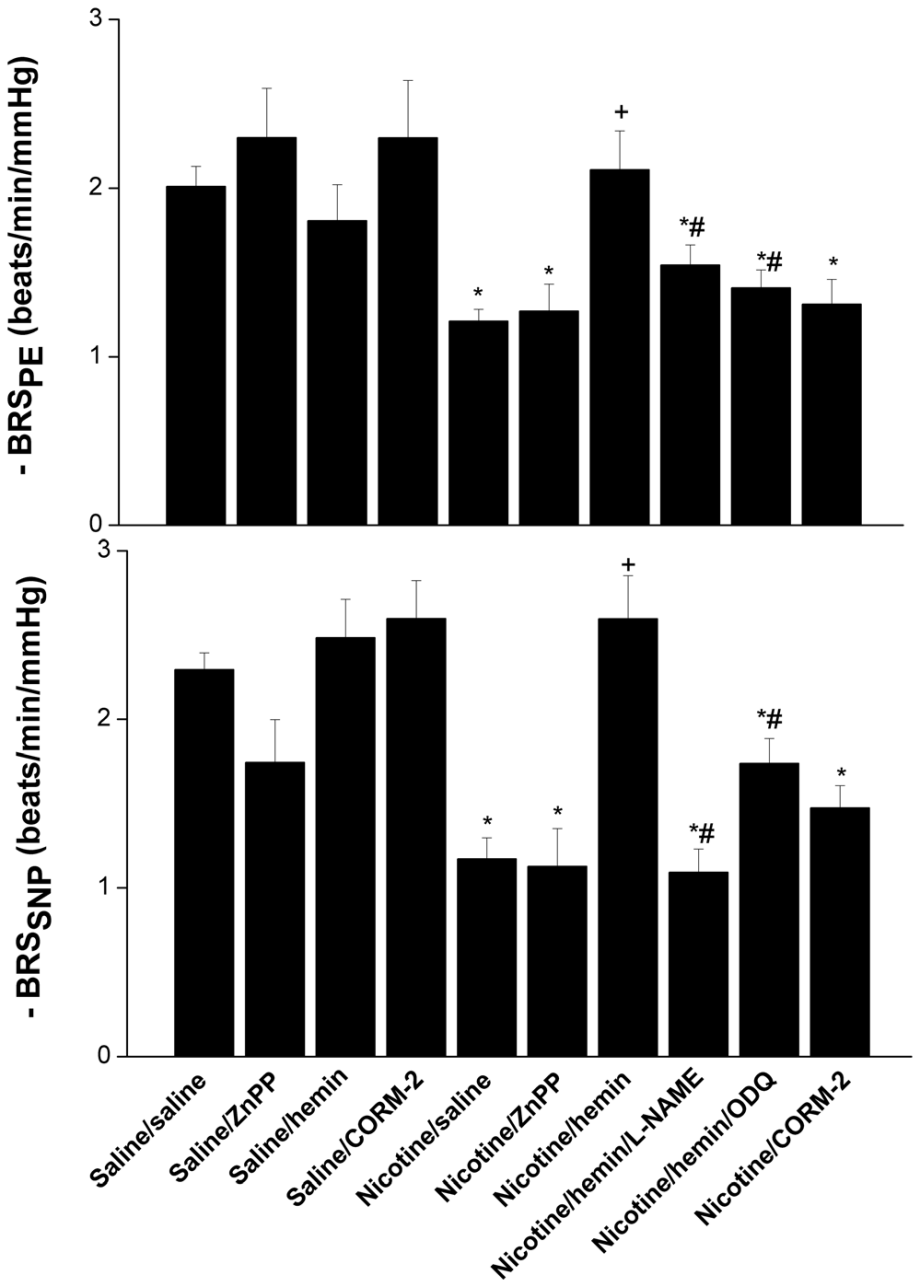
Effect of ZnPP (HO inhibitor), hemin (HO inducer), CORM-2 (CO releasing molecule), or ODQ (guanylate cyclase inhibitor) on baroreflex sensitivity (slopes of baroreflex curves) measured by sodium nitroprusside and phenylephrine in conscious, female rats treated for 14 days with nicotine (2 mg/kg/day, i.p.) or its vehicle (saline). Values are means ± SEM of 6–8 observations. ^*^P<0.05 vs. control (saline/saline), ^+^P<0.05 vs. nicotine, ^#^P<0.05 vs. nicotine/hemin.

The effects of pharmacologic modulators of HO/CO signaling on the nicotine-baroreflex interaction are illustrated in figures 3 and 4. In rats treated chronically with nicotine or saline, HO inhibition by ZnPP (10 mg/kg i.v.) had no effect on baroreflex curves (Fig. 3) or BRS (Fig. 4) measured by SNP or PE. Alternatively, the impairment of baroreflex activity caused by nicotine disappeared in rats treated with hemin (HO inducer, 15 mg/kg, i.v.) as suggested by the shifts in the baroreflex curves towards control (saline-treated) curves (Fig. 3) and the significant increases in the slopes of the regression lines (BRS_SNP_ and BRS_PE_, Fig. 4). This favorable effect of hemin on BRS in nicotine-treated rats was blunted upon concurrent treatment with the NOS inhibitor L-NAME or the guanylate cyclase inhibitor ODQ (Fig. 4). Unlike hemin, the CO releasing molecule CORM-2 (10 mg/kg i.v.) failed to abrogate the nicotine-evoked impairment of baroreflex function (Fig. 4). The treatment of saline –treated rats with hemin or CORM-2 caused no significant changes in baroreflex activity (Fig. 4).

**Figure 5:**
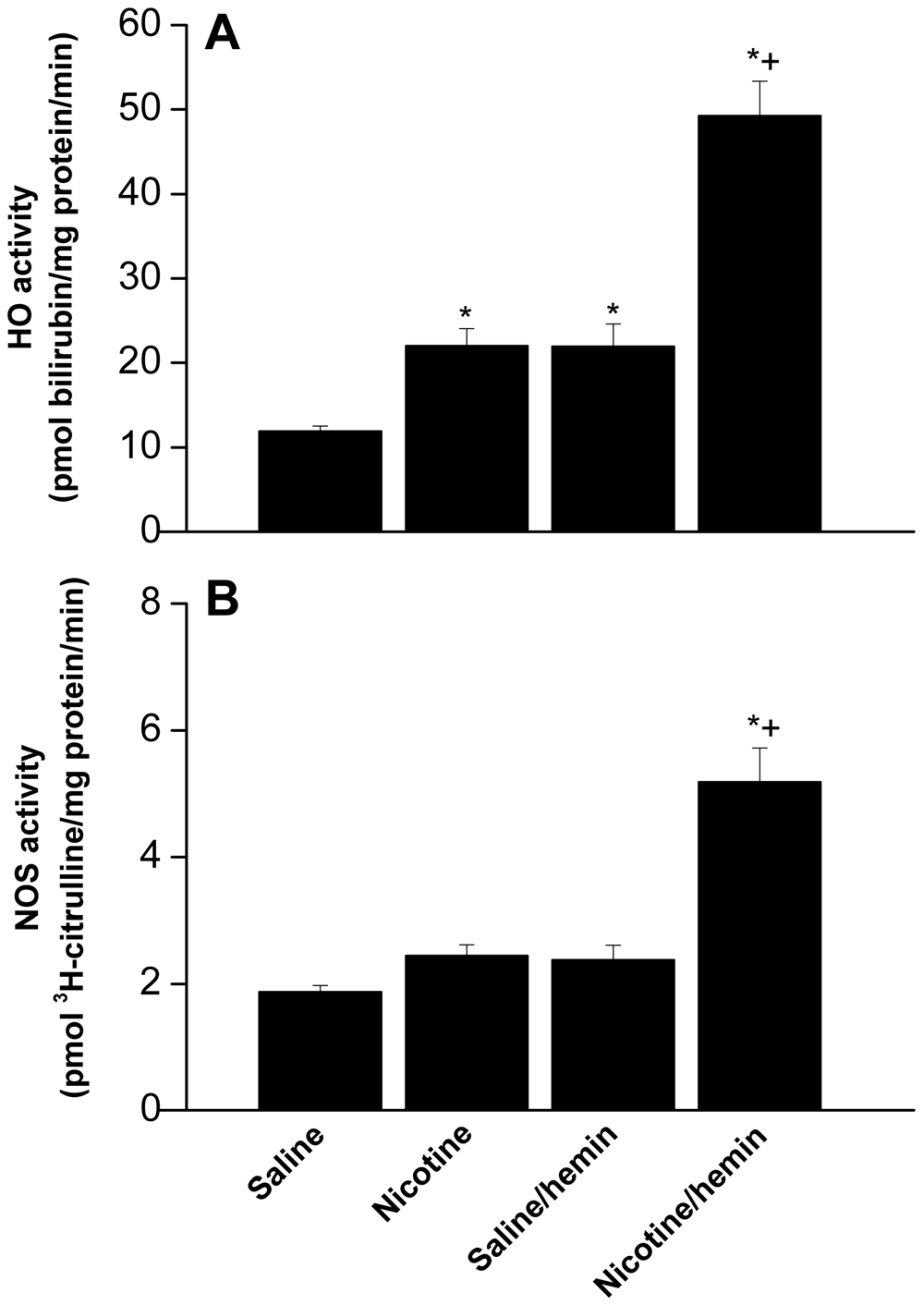
Effect of hemin (15 mg/kg i.v.) on brainstem heme oxygenase (HO, panel A) and nitric oxide synthase (NOS, panel B) activities in female rats treated with nicotine (2 mg/kg/day i.p., 2 weeks) or its vehicle (saline). Values are means ± SEM of 6–8 observations. ^*^P<0.05 vs. “saline” values, ^+^P<0.05 vs. “nicotine” or “saline/hemin” values.

Compared with saline treatment, nicotine significantly increased HO activity in brainstem tissues (12±1 vs. 22±2 pmol bilirubin/mg protein/min, Fig. 5A). A similar increase in HO activity was demonstrated when control rats were treated with i.v. hemin (Fig. 5A). Remarkably greater increases in brainstem HO activity were seen in rats receiving the nicotine/hemin regimen that were significantly higher than the individual effects of the two drugs (Fig. 5A). Further, while the brainstem NOS activity was not affected by nicotine, the administration of hemin to nicotine-treated rats caused significant increases in brainstem NOS activity (Fig. 5B).

#### Effect of bilirubin on the nicotine-baroreflex interaction in female rats

**Figure 6:**
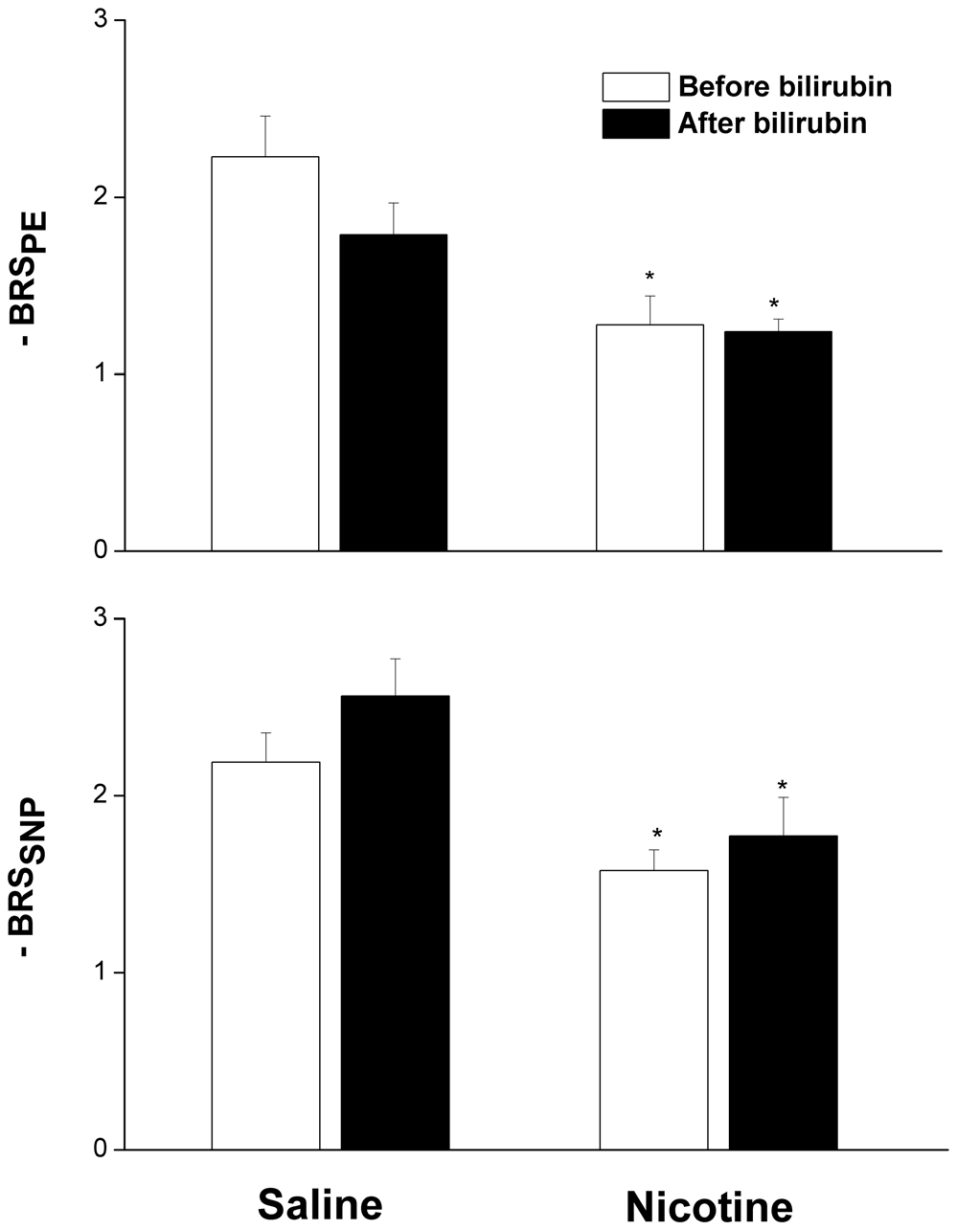
Effect of bilirubin on baroreflex sensitivity (slopes of baroreflex curves) measured by sodium nitroprusside and phenylephrine in conscious female rats treated for 14 days with nicotine (2 mg/kg/day, i.p.) or its vehicle (saline). Values are means ± SEM of 7 observations. ^*^P<0.05 vs. saline.

**Figure 7:**
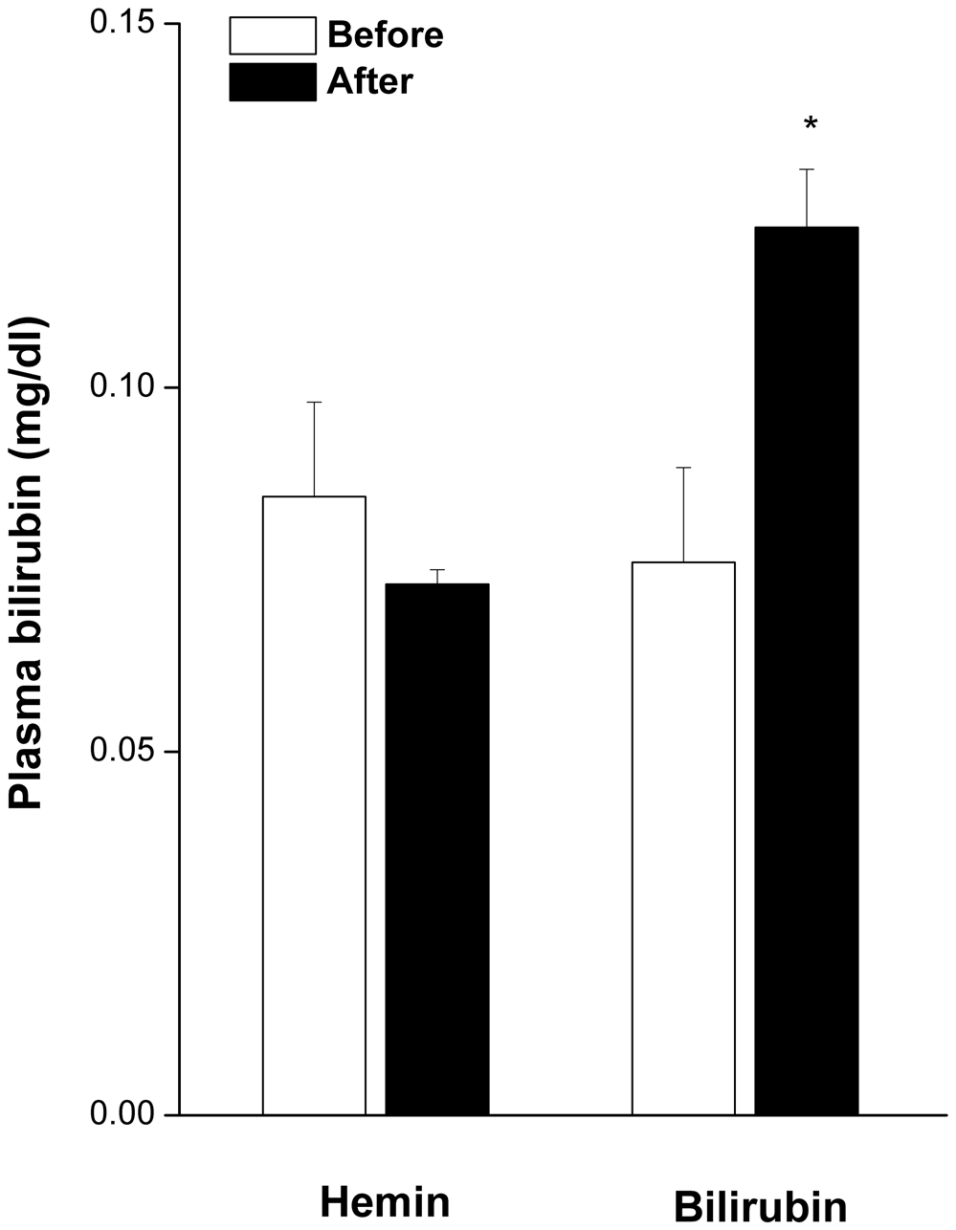
Plasma bilirubin levels in nicotine-treated female rats before and after treatment with hemin or bilirubin. Values are means ± SEM of 7 observations. ^*^P<0.05 vs. respective “before” values.

As shown in figure 6, the i.v. administration of bilirubin (5 mg/kg) caused no changes in reflex chronotropic responses (BRS_PE_ or BRS_SNP_) in control (saline-treated) rats. Moreover, the ability of nicotine (2 mg/kg/day for 14 days) to reduce BRS was still manifest in rats receiving systemic bilirubin (Fig. 6). Plasma bilirubin levels in rats treated with nicotine or saline for 14 days were similar (0.085±0.013 vs 0.080±0.015 mg/dl). Compared with pretreatment levels, plasma bilirubin was significantly increased when measured 15 min after bilirubin administration to nicotine-treated rats (Fig. 7). No change in plasma bilirubin was demonstrated in nicotine-treated rats after the administration of hemin (Fig. 7).

## Discussion

The current study investigated the interesting possibility that the interruption of the NOS/NO and HO/CO pathways accounts for the baroreflex depressant effect of nicotine. The data showed that the interruption of NOS signaling contributes, at least partly, to the inhibitory effect of nicotine on baroreflex gain because (i) pharmacologic NOS inhibition by L-NAME mimicked the baroreflex depressant effect of nicotine, and (ii) concomitant exposure to nicotine and L-NAME elicited no additional reductions in BRS, and (iii) supplementation with the NOS substrate L-arginine mitigated the BRS depressant effect of nicotine. The inability of ZnPP, HO inhibitor, to reduce BRS or to abolish the baroreceptor inhibitory action of nicotine argues against a tonic role for HO/CO in baroreflex control or in the nicotine-baroreflex interaction. Despites, the facilitation of HO activity by hemin increased the baroreflex gain in nicotine-treated rats to levels similar to those of control rats, via facilitating NOS activity.

The NOS-derived NO plays integral role in baroreceptor homeostasis. NOS activity has been demonstrated in central and peripheral sites throughout the cardiac autonomic nervous system, including the receptors and effectors of the baroreflex pathway [9]. Heaton and co-workers [37] showed that NO facilitates the cardiomotor vagal activity via interaction with the pre-/post-ganglionic junction. In the hypothalamus, NO potentiates GABAergic transmission which is the principal inhibitory transmitter in the brain [38] and a key component of the baroreflex arc [39]. These findings prompted us to propose a modulatory role for NOS in the nicotine-evoked impairment of baroreceptor function. The current study presented three main findings in support of this assumption. First, NOS inhibition (L-NAME) in control rats caused shifts in the baroreflex curves and reduced BRS to levels comparable to those seen in nicotine-treated rats. Second, the administration of L-NAME to nicotine-treated rats caused no further reductions in baroreflex activity, suggesting a common baroreflex inhibitory pathway for the two drug interventions (nicotine and L-NAME). Third, enhanced NO generation brought about by L-arginine supplementation normalized baroreflex responsiveness in nicotine-treated rats. These findings together with previous reports including ours that suggested opposite effects for nicotine (inhibition) [7] and NO (facilitation) [37] on cardiomotor vagal activity suggest a pivotal role for inhibition of NOS signaling in the detrimental effect of nicotine on reflex HR responses in female rats.

Because SNP evokes its vasodilatory action via generating NO [40], the use of SNP in the current study for evaluating the effect of nicotine on the reflex tachycardic response and its modulation by NOS might have been inadequate. The possibility, therefore, remains that the apparently opposite vascular effects of the SNP-derived NO and the NOS inhibitor L-NAME [41] might have interfered with the measured baroreflex response. To investigate such assumption, we tested the effect of nicotine on reflex tachycardia generated by hydralazine, a non-NO generating vasodilator. Like its effect on SNP responses, chronic nicotine was found to attenuate the reflex increases in HR that accompanied hydralazine hypotension and this effect of nicotine was blunted after systemic administration of L-arginine. The analogy in the interaction of nicotine with the baroreflex response elicited by SNP and hydralazine made it unlikely that the NO-generating ability of SNP contributed to baroreflex and NOS modulating effects of nicotine.

Given the similarity in the neurobiological and cellular effects of NO and CO, gaseous products of NOS and HO, respectively [8], [9], [13], [14], the issue whether the HO-derived CO also contributes to the interaction of nicotine with baroreflexes was investigated in this study. Our finding that baroreflex responsiveness in control (saline-treated) rats was not altered after HO inhibition by ZnPP suggests no role for heme/HO products in the tonic control of arterial baroreflexes. Likewise, the possibility is unlikely that HO inhibition participated in the impaired baroreflex activity evoked by nicotine treatment because the nicotine/ZnPP-treated rats exhibited significantly attenuated baroreflex gain compared with preparations treated with ZnPP alone. That said, our findings appear to contradict previous reports in which the inhibition of HO activity attenuated baroreceptor function in rats [14], [42], which implies a tonic facilitatory effect for CO on baroreflexes. Several factors may explain the discrepancies between the current and previous studies with regards to the HO modulation of the arterial baroreflex activity. For example, our studies were undertaken in conscious rats in contrast to anesthetized rats in previous studies [14], [42]. Also, the HO inhibitor was injected i.v. in the current study while it was microinjected into the medullary nucleus of the solitary tract in previous studies [14], [42].

Although the present evidence favors no role for HO/CO signaling in baroreflex dysfunction caused by nicotine, we investigated whether the BRS depressant action of nicotine could be mitigated under conditions of enhanced CO generation. The latter was achieved via two different pharmacologic approaches, the use of the HO inducer, hemin, or the CO-releasing molecule, CORM-2. Interestingly, our finding that the adverse BRS effect of nicotine was abolished in rats treated with hemin but not CORM-2 might be interpreted to suggest no role for increased CO generation in the favorable effect of hemin on the nicotine-BRS interaction. Notably, the doses of hemin and CORM-2 employed in the current study have been shown adequate for HO induction and CO generation, respectively [33], [34]. The current finding that the reversal by hemin of the baroreflex depressant effect of nicotine was coupled with remarkable increases in HO and NOS activity in brainstem tissues (see figure 5) establishes the parallelism between the baroreflex and HO/NOS effects of hemin. Notably, the activities of HO and NOS were specifically measured in the brainstem because it encompasses cardiovascular nuclei that critically participate in the central processing of arterial baroreceptor information [13], [15] and in the HO modulation of baroreflex activity [14]. The favorable baroreflex effect of hemin is in line with previous studies in which microinjection of hemin into brainstem barosensitive areas lowered BP and HR and increased neuronal levels of glutamate, effects that are consistent with baroreflex facilitation [14], [42]. The reductions in BP and HR caused by microinjection of glutamate, the chief neurotransmitter of baroreceptor information in NTS, into the solitary tract are eliminated after HO inhibition [15]. Alternatively, the inability of nicotine, when used alone, to alter brainstem NOS activity (Figure 5B) might infer that the interruption by nicotine of downstream effectors of the NOS pathway probably mediated the NOS-dependent baroreflex depressant effect of nicotine. Further, because the whole brainstem was used for the assessment of NOS activity, the data obtained might not precisely reflect changes in NOS activity in medullary cardiovascular nuclei. More studies are needed to investigate this possibility.

HO-1 cleaves heme into CO and biliverdin, which is then reduced enzymatically via biliverdin reductase into bilirubin [43], [44]. Apart from CO, some of the cardiovascular effects of hemin have been attributed to bilirubin, which exhibits a potent antioxidant activity. Considering the parallelism and causal relationship between the antioxidant and baroreflex states [45], we reasoned that an increased generation of bilirubin following hemin administration might contribute to the improved baroreflex responsiveness seen in nicotine/hemin-treated rats. Contrary to our expectations, plasma bilirubin remained unchanged in rats treated with nicotine alone or combined with hemin. Moreover, no changes in reflex chronotropic activity were observed after systemic treatment of nicotine- or saline-treated rats with bilirubin. Notably, the dose of bilirubin employed in the current study has been shown to protect against renal ischemic-reperfusion injury [35]. Obviously, these findings preclude a possible role for bilirubin accumulation in the rectifying action of hemin against baroreflex dysfunction caused by nicotine. It is noteworthy that the catalytic activity of HO-1 also generates cytoprotective products including ferritin and antioxidant molecules [43], [46]. More studies are needed, therefore, to investigate the contribution of these products to the beneficial baroreflex effect of hemin.

It is imperative to comment on the current observation that plasma bilirubin level was not altered by hemin. Importantly, the hemin dose employed in the current investigation (15 mg/kg) matches those used in previous studies and found to increase plasma bilirubin when measured 6 hours following hemin administration [47]. In another study [48], plasma bilirubin was increased after 3 weeks of continuous infusion of hemin via subcutaneous osmotic minipumps. Because plasma bilirubin was measured in our model system 15 min post-hemin administration, it is possible that a longer time might be required for plasma bilirubin to build up to significantly higher levels.

The dynamics and interplay between NOS and HO and their enzymatic products is now of magnificent importance in the understanding of various disease states and potential therapeutic strategies [49]–[51]. Paradoxically, contrasting biological effects for the two systems have also been described [52]. Such discrepancies incited us to investigate whether the enzymatic pathways of NOS and HO mutually interact (synergistically or antagonistically) in their modulation of the nicotine-baroreflex interaction. Our data showed that whereas the inhibition of NOS (L-NAME) or sGC (ODQ) did abolish the ameliorating effect of hemin on baroreflex dysfunction caused by nicotine, the beneficial effect of L-arginine on the nicotine-baroreflex interaction was preserved after HO inhibition by ZnPP. Together, these findings infer that HO might serve as an upstream effector whose activation would lead to consecutive activation of the NOS/sGC signaling and facilitation of the baroreflex response.

In conclusion, the present study provides important and novel information concerning the roles of NOS/HO pathway in the detrimental effect of chronic nicotine on baroreflex responsiveness. The data suggest a key role for impaired NOS, but not HO, signaling in the baroreflex depressant effect of nicotine. The nicotine effect also disappeared in rats treated with the HO inducer hemin through a mechanism that is dependent on NOS, but unrelated to CO or bilirubin accumulation. Clinically, the knowledge gained from the current contribution adds more insight into cellular mechanisms by which nicotine modifies the baroreflex function. The utilization of L-arginine and/or hemin for combating cardiovascular events caused by smoking and related baroreflex dysfunction is warranted.

## Supporting Information

Figure S1 **Pressor and depressor responses elicited by PE and SNP, respectively, and associated reciprocal changes in HR.** Effect of L-arginine (NOS substrate) or L-NAME (NOS inhibitor) on increases or decreases in MAP (panels A and B) elicited by phenylephrine and sodium nitroprusside (1–16 µg/kg, each) respectively, and associated changes in HR (panels C and D) in female rats treated chronically with nicotine (NIC, 2 mg/kg for 14 consecutive days) or saline. BRS values (ΔHR/ΔMAP) determined at equipotent pressor and depressor responses are shown in panels E and F, respectively. Values are means ± SEM of 6–8 observations.^*^P<0.05 versus saline/saline values. ^+^P<0.05 versus nicotine/saline values.
(TIF)

Figure S2 **Effect of chronic nicotine ob baroreflex sensitivity in male rats.** Effect of L-NAME (NOS inhibitor, left panels) or ZnPP (HO inhibitor, right panels) on baroreflex sensitivity (slopes of baroreflex curves) measured by phenylephrine (BRS_PE_) and sodium nitroprusside (BRS_SNP_) in conscious male rats treated for 14 days with nicotine (2 mg/kg/day, i.p.) or its vehicle (saline). Values are means ± SEM of 6 observations. ^*^P<0.05 vs. respective “before L-NAME” or “before ZnPP” in the saline group, ^+^P<0.05 vs. respective “after ZnPP” values in the saline group.
(TIF)

File S1 **(i) Role of altered pressor and depressor responsiveness in the nicotine-baroreflex interaction, and (ii) Effect of nicotine on baroreflexes in male rats.**
(DOCX)
