# Supplementary material for: Impairment of Nitric Oxide Synthase but Not Heme Oxygenase Accounts for Baroreflex Dysfunction Caused by Chronic Nicotine in Female Rats
Source: PLoS One. 2014 May 28;9(5):e98681. doi: 10.1371/journal.pone.0098681 (PMC4037226; doi:10.1371/journal.pone.0098681)
Supplement: File S1 — (i) Role of altered pressor and depressor responsiveness in the nicotine-baroreflex interaction, and (ii) Effect of nicotine on baroreflexes in male rats. (DOCX) [file pone.0098681.s003.docx]

**Protocols**

**Role of altered MAP responsiveness to PE or SNP in the evoked baroreflex response.** To eliminate any role for the altered MAP responsiveness to PE or SNP caused by the modulators of NOS activity in the associated BRS changes, HR responses to similar increases (PE, ~ 40 mmHg) or decreases (SNP, ~ -20 mmHg) in MAP were computed for individual rats, regardless of the doses of vasoactive agents employed. The ratio ∆HR/∆MAP was taken as a measure of BRS.

**Effect of nicotine on baroreflex gain in male rats.** This experiment investigated whether the interaction of chronic nicotine with reflex HR control seen in female rats and its modulation by the NOS/HO signaling could be replicated in male rats. Four groups of conscious male rats (n=6 each), pre-instrumented with indwelling femoral catheters, were used to determine the effect of 14-day treatment with the 2 mg/kg/day dose of nicotine or saline on reflex chronotropic responses to i.v. injections of PE or SNP (1-16 μg/kg each) before and 15 min post i.v. administration of 10 mg/kg of L-NAME or ZnPP.

**Results**

**BRS at equipotent pressor and depressor responsiveness**

Compared with respective control values, i.v. L-NAME, but not L-arginine, significantly enhanced the depressor responses elicited by SNP in rats treated chronically with nicotine or saline (Fig. S1-B). On the other hand, neither L-NAME nor L-arginine affected the pressor actions of PE (Fig. S1-A). At equipotent pressor (PE, ~ 40 mmHg) or depressor responses (SNP, ~ -20 mmHg), BRS (∆HR/∆MAP) was significantly reduced by nicotine or L-NAME treatment (Fig. S1-E, S1-F). The supplementation with L-arginine had no effect on BRS in saline-treated rats, but reversed the attenuated BRS demonstrated in nicotine-treated rats (Fig. S1-E, S1-F).

**NOS/HO modulation of the baroreflex effects of nicotine in male rats**

As shown in figure S2, the 2-week treatment of male rats with nicotine (2 mg/kg/day i.p.) reduced reflex chronotropic responses as indicated by the significant reductions in the slopes of the baroreflex curves (BRS_PE_ and BRS_SNP_) compared with saline-treated rats. Both BRS_PE_ and BRS_SNP_ were reduced after the enzymatic inhibition of NOS by L-NAME in saline-treated, but not in nicotine-treated, male rats (Fig. S2). On the other hand, no BRS changes were seen in both groups of rats (saline or nicotine) after HO inhibition by ZnPP (Fig. S2).

**Discussion**

Because the current observation that the depressor effect of SNP was enhanced in rats with inhibited NOS (Fig. S1), it is likely that the changes caused by modulators of NOS activity (L-NAME or L-arginine) in vascular responsiveness might have contributed to the evoked alterations in baroreflex activity. To investigate this possibility, BRS was computed using reflex HR responses elicited by equipotent depressor and pressor responses [1]. The data showed that BRS changes caused by L-NAME or L-arginine in nicotine or saline-treated rats were replicated when equipotent MAP responses were considered. It is unlikely, therefore, that the altered MAP responsiveness to NOS modulators has contributed to BRS changes caused by these drugs and their interaction with nicotine.

Interestingly, because the baroreflex depressant effects of nicotine and their dependence on NOS, but not HO, activity were reproduced in male rats, our findings argue against any gender specificity in the interaction of chronic nicotine with the baroreflex HR control.

**References**

1. El-Mas MM, Afify EA, Omar AG, Sharabi FM (2002) Cyclosporine attenuates the autonomic modulation of reflex chronotropic responses in conscious rats. Can J Physiol Pharmacol 80: 766-776.
